# Contrasting growth responses to aluminium addition among populations of the aluminium accumulator *Melastoma malabathricum*

**DOI:** 10.1093/aobpla/plaa049

**Published:** 2020-09-11

**Authors:** Khairil Mahmud, David F R P Burslem

**Affiliations:** 1 Department of Crop Science, Faculty of Agriculture, Universiti Putra Malaysia (UPM), Seri Kembangan, Selangor, Malaysia; 2 School of Biological Sciences, University of Aberdeen, AB242UU Aberdeen, Scotland, UK; 3 School of Agricultural Science, Faculty of Bioresources and Food Industry, Universiti Sultan Zainal Abidin, Besut Campus, Besut, Terengganu, Malaysia

**Keywords:** Aluminium accumulator, *Melastoma malabathricum*, functional trait, relative growth rate, Peninsular Malaysia

## Abstract

Aluminium (Al) hyper-accumulation is a common trait expressed by tropical woody plants growing on acidic soils. Studies on Al accumulators have suggested that Al addition may enhance plant growth rates, but the functional significance of this trait and the mechanistic basis of the growth response are uncertain. This study aimed to test the hypothesis that differential growth responses to Al among populations of an Al accumulator species are associated with variation in biomass allocation and nutrient uptake. We conducted two experiments to test differential responses to the presence of Al in the growth medium for seedlings of the Al accumulator shrub *Melastoma malabathricum* collected from 18 populations across Peninsular Malaysia. Total dry mass and relative growth rate of dry mass were significantly greater for seedlings that had received Al in the growth medium than for control plants that did not receive Al, but growth declined in response to 5.0 mM Al addition. The increase in growth rate in response to Al addition was greater for a fast-growing than a slow-growing population. The increase in growth rate in response to Al addition occurred despite a reduction in dry mass allocation to leaves, at the expense of higher allocation to roots and stems, for plants grown with Al. Foliar concentrations of P, K, Mg and Ca increased in response to Al addition and the first axis of a PCA summarizing foliar nutrient concentrations among populations was correlated positively with seedling relative growth rates. Some populations of the Al hyper-accumulator *M. malabathricum* express a physiological response to Al addition which leads to a stimulation of growth up to an optimum value of Al in the growth medium, beyond which growth declines. This was associated with enhanced nutrient concentrations in leaves, which suggests that Al accumulation functions to optimize elemental stoichiometry and growth rate.

## Teaser

The relative growth rate of dry mass was significantly greater for populations of *M. malabathricum* seedlings that had received Al in the growth medium than for control plants. The increase in growth rate in response to Al addition occurred despite a reduction in dry mass allocation to leaves, at the expense of higher allocation to roots and stems, for plants grown with Al. Foliar concentrations of P, K, Mg and Ca increased in response to Al addition and foliar nutrient concentrations among populations were correlated positively with seedling relative growth rates. Some populations of *M. malabathricum* express a physiological response to Al addition which leads to a stimulation of growth up to an optimum value of Al in the growth medium, beyond which growth declines.

## Introduction

The toxicity of high soil Aluminium (Al) concentrations to many plants is an important limitation to crop production on acidic soils globally (Ryan *et al.* 1993; Doncheva 2005; R’bia *et al.* 2011). For this reason, understanding the mechanisms of Al uptake and toxicity has been a major focus of plant physiological research (Godbold *et al.* 1988; Watanabe *et al.* 1997; Barceló and Poschenrieder 2002). However, plants that are able to tolerate and even accumulate Al are particularly interesting as potential model organisms for understanding the physiological constraints to plant growth and productivity on acid soils. Al accumulators were originally defined as plants that contain more than 1.0 mg Al g^-1^ dry mass (Chenery 1948), although more recent surveys of Al concentrations in plant tissues suggest that this threshold may be dependent on biogeographic origin and growing conditions (Metali 2010; Metali *et al.* 2012). Al accumulators occur in approximately 60 angiosperm families that are distributed broadly among eudicots and monocots, as well as some ferns and mosses, which suggests that the Al accumulation trait has evolved many times (Chenery 1948; Jansen 2002). These species contain tissue Al concentrations that are far in excess of those found in a majority of plants, without suffering phytotoxic effects (Jansen 2002; Rascio and Navari-Izzo 2011). Most Al accumulator plants have been recorded from tropical biomes and are associated with the acidic soils of these regions (Jansen *et al.* 2002; Osaki *et al.* 2003; Metali *et al.* 2012).

Although the phytotoxic effects of Al on plants dominate physiological research, there have also been persistent reports of positive effects of Al addition at low concentrations on the growth of some plants grown in nutrient solutions. These stimulatory effects of Al have been demonstrated for non-Al accumulators native to acid soils, such as *Miscanthus sinensis* (Yoshii 1937) and *Eucalyptus gummifera* (Mullette 1975), as well as Al accumulators such as *Melastoma malabathricum* (Osaki *et al.* 1997; Watanabe *et al.* 1998, .2005; Metali 2010), *Camelia sinensis* (Konishi *et al.* 1985; Fung *et al.* 2008; Hajiboland *et al.* 2013; Tolra *et al.* 2020), *Vochysia tucanorum* (de Souza *et al*. 2017), *Callisthene fasciculata* (de Souza *et al.* 2020) and *Symplocos paniculata* (Schmitt *et al.* 2016). A positive growth response to Al addition represents a paradox considering the highly toxic effects of Al observed in most plants and especially crops (Hajiboland *et al.* 2013; Poschenrieder *et al.* 2015).

Several mechanisms have been proposed to explain this positive growth response to Al addition. One proposal is that Al efflux from plant roots ameliorates H^+^ toxicity for plants growing in acid soils (Osaki *et al.* 1997; Kidd and Proctor 2001; Delhaize *et al.* 2012), but the inherent tolerance of many Al-responsive plants to low pH soils undermines support for this hypothesis (Hajiboland *et al.* 2013). Alleviation of an inhibitory effect of excess P on plant growth (Clark 1977) is equally unlikely considering the low P concentrations in uncultivated soils where wild Al accumulators typically occur (Metali *et al.* 2012). An alternative hypothesis is that Al addition stimulates the uptake of N, P or K and growth is thus stimulated by alleviation of deficiencies of these nutrients (Konishi *et al.* 1985; Osaki *et al.* 1997; Watanabe *et al.* 2005). Although there appears to be evidence for this hypothesis (Osaki *et al.* 1997), later experiments focussing on P uptake failed to support it (Watanabe and Osaki 2001). This hypothesis is also difficult to interpret when plants are grown in nutrient solutions containing high concentrations of these elements (e.g. Watanabe and Osaki 2001; Watanabe *et al.* 2005). Similarly, protection against Mn toxicity (Clark 1977) would only be a credible mechanism of growth stimulation by Al for plants growing in solutions containing high Mn concentrations, which is not typical for studies of this type. In tea (*Camelia sinensis*), low concentrations of Al in nutrient solutions stimulate increased rates of stomatal conductance and photosynthesis, which would provide a direct explanation for the positive effect on growth (Hajiboland *et al.* 2013).

The tropical Southeast Asian shrub *Melastoma malabathricum* is emerging as a model species for ecophysiological studies on Al accumulation (Osaki *et al.* 1997; Watanabe *et al.* 1998; Metali *et al.* 2015; Khairil and Burslem 2018). This species has shown enhanced growth and root activity and increased uptake of N, P, K, Ca and Mg in response to addition of Al to nutrient solutions (Watanabe and Osaki 2001; Watanabe *et al.* 1997, 2005).

It is a widespread species in tropical Asia and occupies a diverse range of habitats and soil conditions (Watanabe *et al.* 1998, 2005; Khairil and Burslem 2018). We have shown elsewhere that the expression of foliar Al accumulation varies among populations of *M. malabathricum*, and that these differences are positively correlated with total soil concentrations of N, Ca and Mg, but unrelated to soil total or exchangeable Al concentrations for populations in the wild (Khairil and Burslem 2018). In a solution culture experiment, Al addition increased foliar concentrations of P, K, Ca and Mg (Khairil and Burslem 2018). In this paper, we test the hypothesis that differential growth responses to Al addition among populations of this species are related to their capacity for nutrient uptake. We compare these results for tissue chemistry to the alternative hypothesis that growth responses to Al addition are driven by increased biomass allocation to leaves. The following specific questions were addressed.

Do *M. malabathricum* seedlings respond positively to the addition of Al to the growth medium?
Does the extent of growth stimulation vary among progeny derived from different populations of *M. malabathricum*?
Is growth stimulation by Al addition associated with changes in biomass allocation and/or changes in leaf tissue chemistry?

## Methods

### Study species

The study species was the tropical shrub *Melastoma malabathricum* (Melastomataceae), which is a known Al accumulator plant (Chenery 1948; Jansen 2003; Watanabe *et al.* 2005; Khairil and Burslem 2018). *M. malabathricum* occurs from islands in the Indian Ocean to South and South-East Asia, China, Taiwan, Australia and the South Pacific Ocean and is found in a range of natural vegetation types, as well as wasteland, secondary forest and roadsides (Jansen *et al.* 2002; Watanabe *et al.* 2005). In some countries, including Malaysia, the leaves and roots of *M. malabathricum* are reported to be useful for medicinal purposes (Sharma *et al.* 2001; Joffry *et al.* 2012).

### Plant sampling

Fruits of *M. malabathricum* were collected from 18 populations across Peninsula Malaysia (see **Supporting Information—Figure S1**) growing in a range of habitat types and elevations, spanning 2–450 m a.s.l., in December 2013 and January 2014 (Khairil and Burslem 2018). A total of 10–12 fruits from at least three individuals (range 3–5 individuals) were collected per population and mixed together to create a bulk sample for each population. The seeds were extracted from the partly opened fleshy fruits in distilled water, rinsed with distilled water several times, then filtered and left to air-dry in an air-conditioned laboratory at the Universiti Sultan Zainal Abidin, Malaysia. Air-dried seeds were transported to the University of Aberdeen, UK, for experimental work.

The seeds were soaked in 5 % bleach solution for 3 min and then rinsed three times for at least 3 min with sterilized distilled water. Three seeds from each population were then sown together on the surface of Daishin agar (0.5 g agar/100 mL with 50 % Hoagland nutrient solution) in sterilized 0.5 µL Eppendorf tubes (for the composition of nutrient solution [see Supporting Information—Table S2 of Khairil and Burslem 2018). The bottom 3 mm of the Eppendorf tubes had been removed to enable the growth of roots into a nutrient solution. Each population was represented by 24 Eppendorf tubes (72 seeds per population). Thereafter, the 432 tubes were suspended in groups of six in sterilized boxes (dimensions 12 × 8 × 7 cm) containing 50 % Hoagland nutrient solution with each box containing three tubes of each of two populations. The boxes were divided equally between two growth chambers both set to deliver a temperature of 27 °C and 12/12 h light/dark photoperiod with an irradiance of 200–250 μmol m^–2^ s^–1^. The pH of the nutrient solutions was checked daily and adjusted to 4.0 following Watanabe and Osaki (2002) using 0.1 M NaOH or 0.1 M HCl, and the nutrient solutions were renewed weekly throughout the growing period. The seeds germinated after 7–10 days, and 14 days after sowing the seedlings were thinned to one per Eppendorf tube by randomly selecting excess surviving individuals for removal.

Twenty-eight days after sowing, containers containing half the seedlings per population were randomly selected and the seedlings were harvested, oven-dried at 60 °C and weighed (harvest 1). Half of the remaining seedlings per population were randomly selected to receive Al in the form of 1.0 mM AlC_3_ added to the nutrient solution. Six individuals in two boxes per population in each treatment were distributed equally between the two growth chambers. The boxes were rerandomized weekly within the growth chambers and all remaining seedlings were then harvested after 56 days, dried and weighed (harvest 2).

To determine elemental concentrations in plant leaves, a 0.5–1.0 cm^2^ fragment of the lamina tissue was removed from the leaf margin for three randomly selected individuals per population × treatment combination at harvest 2. This material was cut in transverse section, washed in deionized water and placed in a 50 µL Teflon tube. The samples were then dried in an oven at 88 °C for 20–22 h and weighed before being digested using 70 % nitric acid (HNO_3_) and analysed for Al, Ca, Mg, K and P by inductively coupled plasma mass spectrometry (ICP-MS) (NexION 300D, ICP Mass Spectrometer, PerkinElmer, USA). The dry mass of samples used for analysis was added to the remaining leaf dry mass to derive the total leaf dry mass per plant.

In a second experiment, the two populations of *M. malabathricum* which had the highest and lowest relative growth rates in the first experiment were selected for detailed characterization of their growth response to a range of Al concentrations in the growing medium. A total of 360 seeds per population were germinated separately in 120 Eppendorf tubes (60 per population) on the surface of 50 % Hoagland agar from which the lowest 0.5 cm had been removed to allow root growth into surrounding nutrient solution as above. The tubes were distributed among 30 sterilized containers (dimensions 12 × 8 × 7 cm) containing 50 % Hoagland solution. The containers were divided equally between two growth chambers with identical growing conditions and adjustments to the nutrient solution pH as in Experiment 1.

The seeds germinated after 7–9 days, and 14 days after sowing the seedlings were thinned to one seedling per Eppendorf tube by randomly selecting excess surviving individuals for removal. Boxes were re-randomized weekly within each growth chamber. Four weeks after germination was complete (35 days from sowing), half of the seedlings were harvested, oven-dried for 5 days at 60 °C and the dry mass of the seedlings measured. The 30 remaining seedlings from each population were suspended, one per container, in nutrient solutions comprising 50 % Hoagland solution amended with the addition of 0, 0.5, 1.0, 2.0 or 5.0 mM AlCl_3_ to achieve six replicates per Al treatment for each population. The 60 containers were distributed randomly between the two growth chambers and their locations re-randomized weekly within growth chambers. Four weeks after the start of the Al treatments (9 weeks from sowing), all seedlings were harvested by dividing each plant into fractions representing roots, stems and leaves before oven-drying them at 60 °C for 5 days. The root, stem and leaf dry mass of each individual was determined separately and biomass allocation represented as the dry mass ratios (dry mass of a plant part divided by total plant dry mass), for leaves (leaf mass ratio, LMR), stems and petioles (stem mass ratio, SMR) and roots (root mass ratio, RMR) at the final harvest (Hunt 1982).

### Data processing and statistical analysis

The relative growth rate of dry mass (RGR) was calculated based on Hunt (1982):

$$\begin{array}{l} RGR\;=\;\left(loge\;W_{2}-loge\;W_{1}\right)/\;\left(t_{2}-t_{1}\right) \end{array}$$

where *W*_2_ is the final total plant dry masses (g) of each individual at the second harvest conducted at time *t*_2_ and *W*_1_ represents the mean total dry mass of the population from which that individual was derived at the first harvest conducted at time *t*_1_.

Two-way analysis of variance (ANOVA) models were fitted to data on total dry mass at the final harvest, RGR and mass allocation variables as well as the foliar Al and nutrient concentrations to determine the significance of differences among populations, the Al treatments (Al+ vs. Al− in the first experiment, or the five Al treatments used in Experiment 2) and the interaction between population and Al treatments. The major trends across the multivariate data-sets of foliar concentrations of five elements (Al, P, K, Ca and Mg) and biomass allocation (LMR, SMR and RMR) were summarized using Principal Components Analyses (PCA) on centred and standardized data for plants derived from the Al+ and Al− treatments separately in Experiment 1, and Pearson correlations were used to determine whether scores along the first two PC axes were related to population mean values of dry mass, relative growth rate or the percentage change in these metrics in response to Al addition. All analyses were conducted using R version 3.3.1 (R Development Core Team 2016) using the aov function in the ggplot library for ANOVA and the prcomp() function in the ggbiplot library for PCA analyses.

## Results

### Effects of Al addition on seedling growth

**Figure 1.**
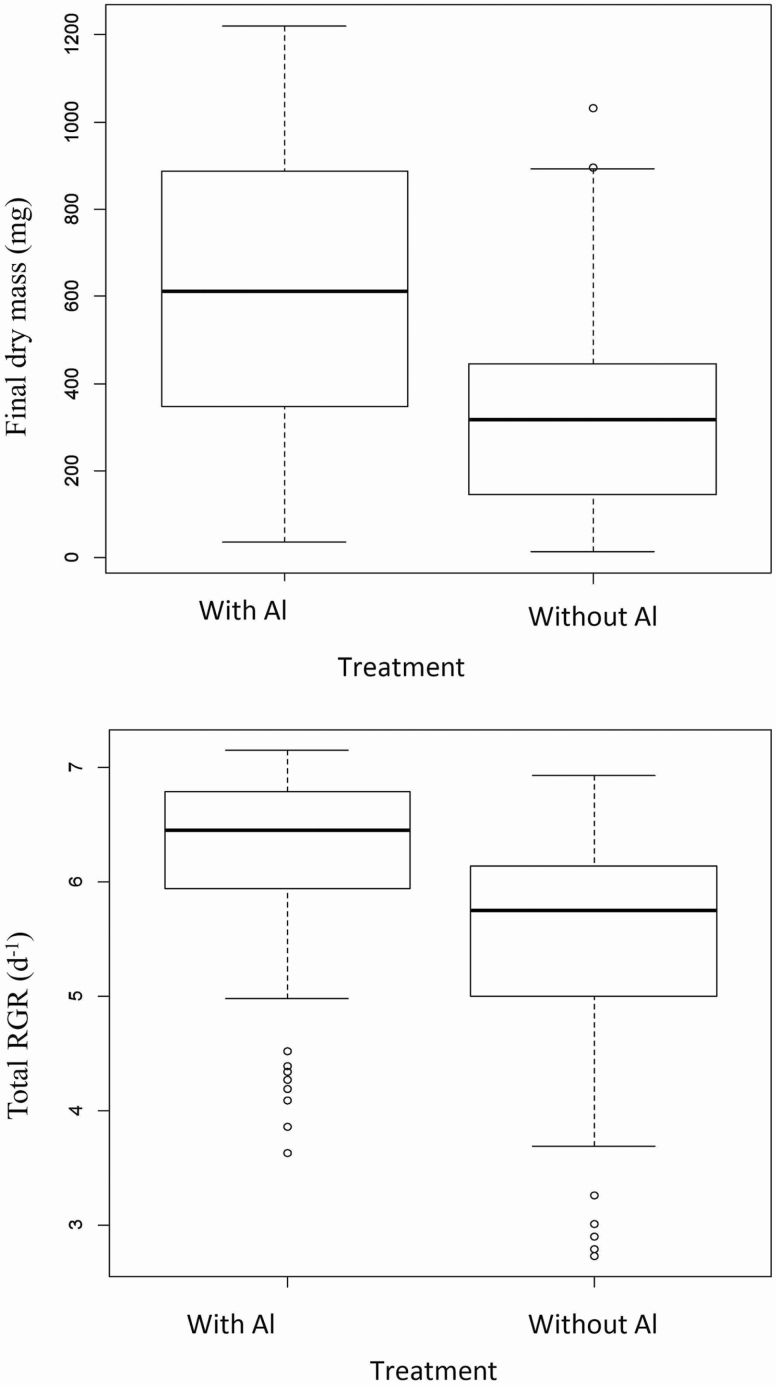
Boxplots of final dry mass (mg) for seedlings (top panel) and mean (±SEM) relative growth rate (d^−1^) (lower panel) derived from 18 populations of *Melastoma malabathricum* across Peninsular Malaysia and grown in the absence or presence of 1 mM Al^3+^ in a nutrient solution.

In Experiment 1, mean seedling dry mass after 56 days and RGR from days 28 to 56 following Al addition increased by 94 % (*F* = 82.2, *P* < 0.001) and 14 % (*F* = 47.2, *P* < 0.001), respectively, in response to the addition of Al to the nutrient solution (Fig. 1, for two-way ANOVA output, **see ****Supporting Information—Tables S1**–S3). Seedling dry mass and RGR also differed significantly among the 18 populations of *M. malabathricum* (*F* = 7.5, *P* < 0.001; *F* = 6.1, *P* < 0.001, for dry mass and RGR, respectively), but there was no evidence of a significant interaction of Al treatment and population for either growth measure.

**Figure 2.**
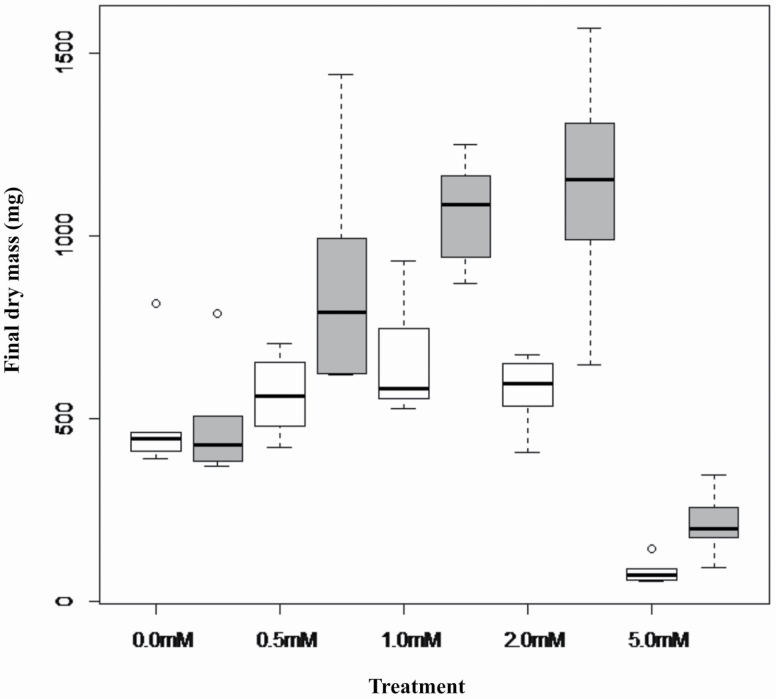
Boxplots of mean final dry mass *M. malabathricum* seedlings of slow-growing (white) and fast-growing (dark) populations after growth for 28 days in nutrient solutions containing 0 mM, 0.5 mM, 1.0 mM, 2.0 mM and 5.0 mM AlCl_3_.

**Figure 3.**
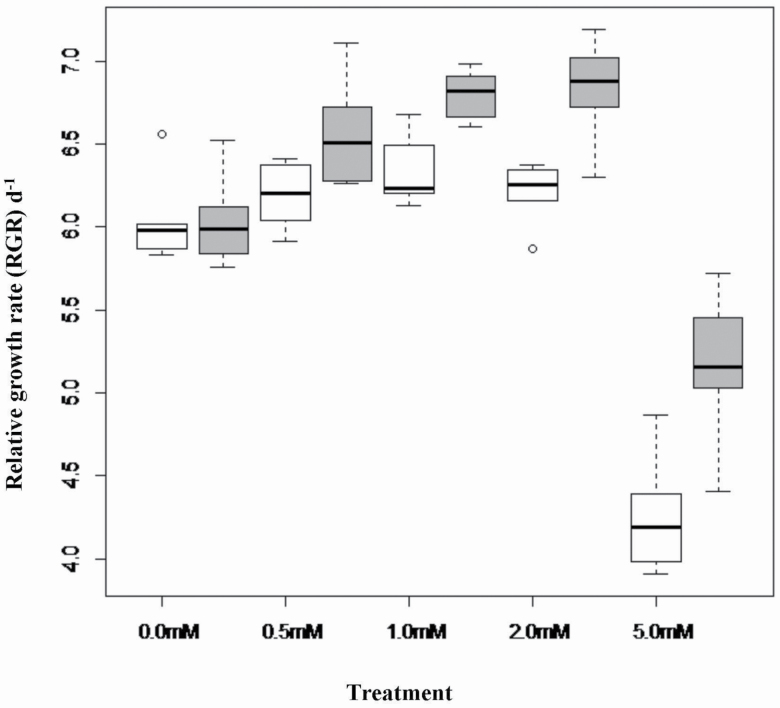
Boxplots of final dry mass (mg) of seedlings of slow-growing (white) and fast-growing (dark) populations of *M. malabathricum* after growth for 28 days in nutrient solutions containing 0 mM, 0.5 mM, 1.0 mM, 2.0 mM or 5.0 mM AlCl_3_.

In Experiment 2, mean total dry mass (Fig. 2) and RGR (Fig. 3) differed significantly between the two populations and were greater for plants grown in solutions containing Al in concentrations up 2.0 mM AlCl_3_, and the response to Al concentration treatments differed significantly between the two populations **[see ****Supporting Information—Tables S4**  **and**  **S5****]**. When Al was absent from the nutrient solution, dry mass and RGR of the two populations were similar, but both growth metrics increased in response to an increase in Al concentration up to maximum values for solutions containing 1.0 mM Al for the slow-growing population or 2.0 mM Al for the fast-growing population. For both populations, dry mass and RGR declined significantly below that of the no-addition control for seedlings grown in solutions containing 5.0 mM Al (Figs 2 and 3). The growth response to Al addition was much greater for seedlings of the faster growing population, which led to substantially higher values of these growth metrics in all Al addition treatments and maintenance of a growth response to Al up to the 2.0 mM Al treatment, despite the similar mean values for growth in the no-addition control treatment.

### Biomass allocation

**Figure 4.**
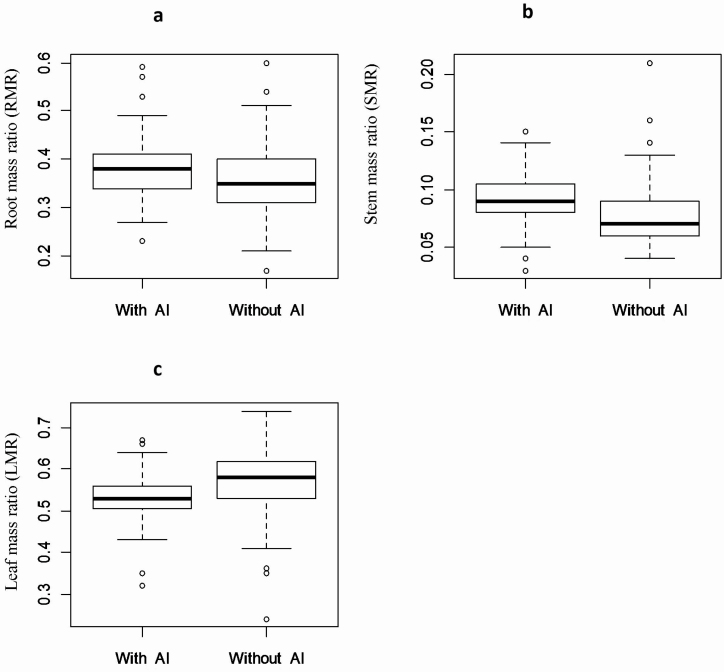
Boxplots of root mass ratio (RMR) **(A)**, stem mass ratio (SMR) **(B)** and leaf mass ratio (LMR) **(C)** of seedlings derived from 18 populations of *M. malabathricum* across Peninsular Malaysia and grown for 28 days in the absence or presence of 1 mM Al^3+^ in a nutrient solution.

Across the 18 populations compared in Experiment 1, mean root mass ratio and stem mass ratio increased by 2.4 % (*F* = 6.99, *P* < 0.01) and 1.0 % (*F* = 4.51, *P* < 0.01), respectively, in response to the addition of 1.0 mM Al to the nutrient solution (Fig. 4; **see ****Supporting Information—Table S6**), while mean leaf mass ratio decreased by 3.4 % (*F* = 14.2, *P* < 0.001). All three mass ratios also differed significantly among populations, but the interaction of Al treatment and population was not significant in Experiment 1 **[see ****Supporting Information—Table S6****]**.

**Figure 5.**
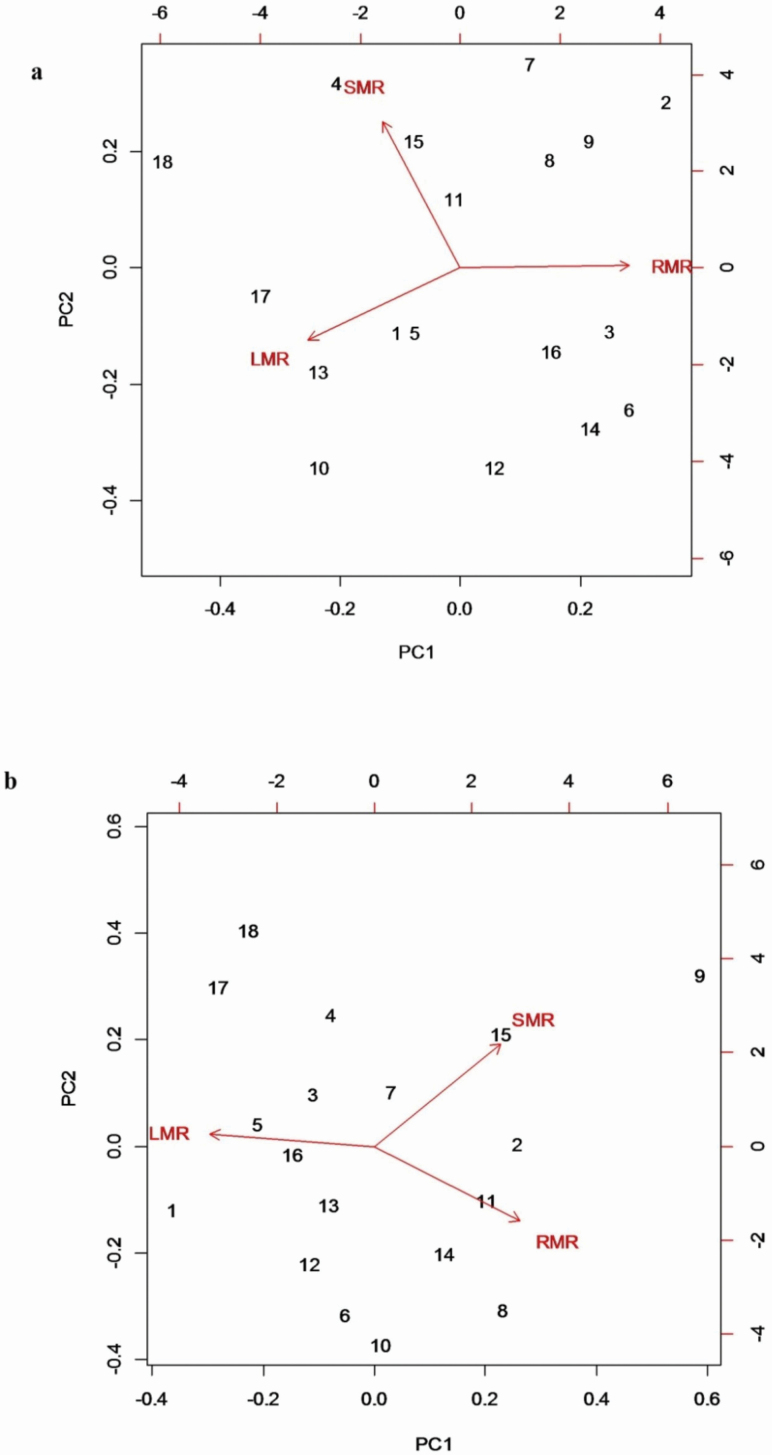
Biplots showing the distribution of 18 populations of *M. malabathricum* along principal component axes 1 and 2 from PCAs summarizing variation in biomass allocation variables (RMR, SMR and LMR) for seedlings grown for 28 days either (A) with Al addition or (B) without Al addition. In (A) PC1 and PC2 accounted for 66.8 and 32.7 % of the total variation, respectively, while in (B) PC1 and PC2 account for 78.3 and 21.5 % of the total variation, respectively.

A PCA based on population mean values of biomass allocation variables (RMR, SMR and LMR) for seedlings grown with Al addition uncovered a single axis explaining 66.8 % of the variation in the data and a second axis explaining 32.7 % of the variation (Fig. 5A; **see ****Supporting Information—Table S7**). The equivalent PCA for seedlings grown without Al addition displayed a first PC axis explaining 78.3 % of the variation and a second axis explaining a further 21.5 % of the variation (Fig. 5B; **see ****Supporting Information—Table S8**). In both cases, the dominant first axis largely represented differential allocation to roots vs. leaves, while the secondary axis represented differential allocation to stem mass. However, there were no significant correlations between axis scores along the first two principal components for biomass allocation and percentage stimulation of either dry mass (PC1: *r* = 0.194, *P* = 0.44; PC2: *r* = 0.422, *P* = 0.081) or relative growth rate (PC1: *r* = 0.409, *P* = 0.09; PC2: *r* = 0.301, *P* = 0.22) among populations in response to Al addition. Furthermore, there were no significant correlations between these mass allocation ratios and foliar Al concentrations among populations for seedlings that had been grown in the presence of Al (*P* > 0.05).

For the two populations compared in Experiment 2, mean values of root mass ratio and stem mass ratio increased significantly in response to successive increases in the concentration of Al in the nutrient solution, while values of leaf mass ratio declined **[see ****Supporting Information—Figures S2–S4****]**. However, there were no significant interactions observed between population and Al treatments, which supports the result from the first experiment that the response to Al treatments in terms of biomass allocation are similar in magnitude among populations **[see ****Supporting Information—Table S9****]**.

### Foliar Al and nutrient concentrations

**Figure 6.**
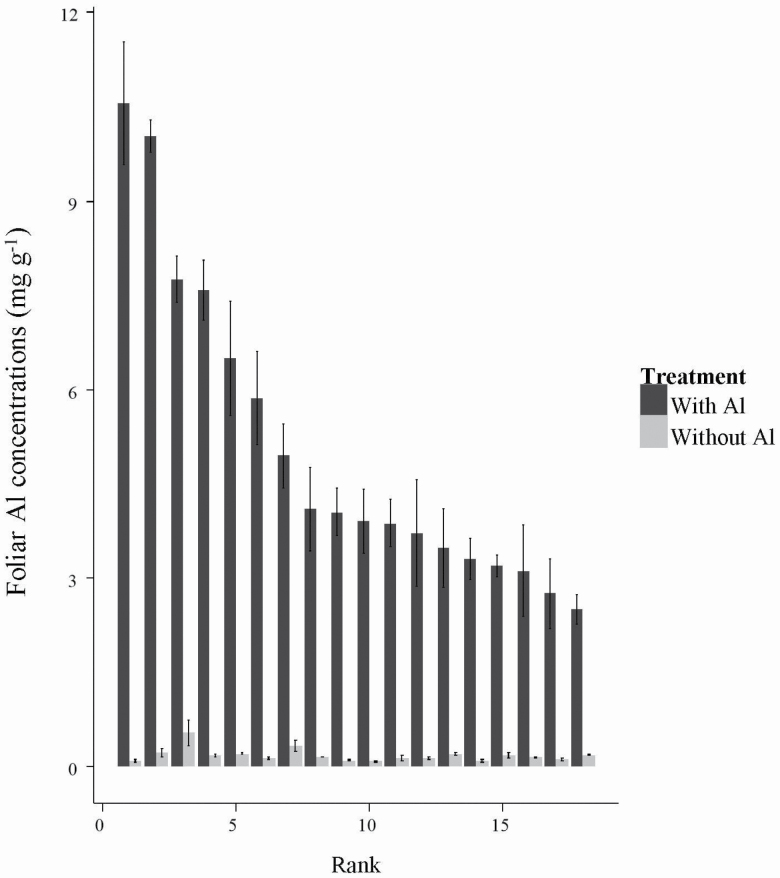
Mean (± SEM) foliar Al concentrations in seedlings derived from 18 populations of *Melastoma malabathric*um collected in Peninsular Malaysia and grown for 28 days in the absence (grey bars) or presence (black bars) of 1 mM Al^3+^ in a nutrient solution. Populations are ranked from highest to lowest values of foliar Al concentration in the +Al treatment.

**Figure 7.**
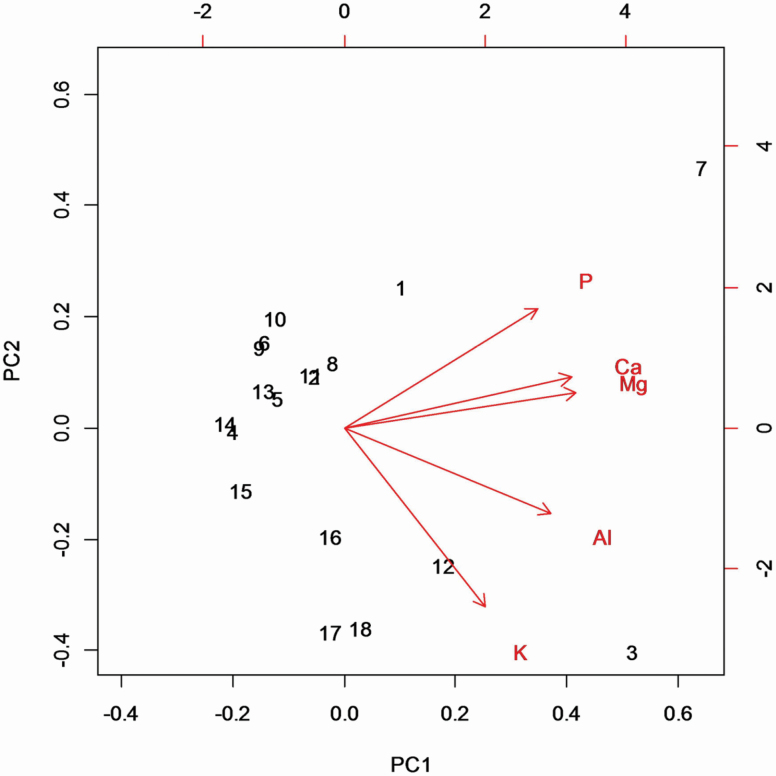
Biplot showing the distribution of 18 populations of *M. malabathricum* along principal component axes 1 and 2 from a PCA summarizing variation in foliar Al, P, K, Ca and Mg concentrations for seedlings grown with Al addition (1.0 mM AlCl_3_). PC1 and PC2 accounted for 71.6 and 20.0 % of the total variation, respectively. The arrows show the loadings of each variable on the first two principal component axes.

In the absence of Al addition, mean (±SEM) foliar Al concentration across the 18 populations of *M. malabathricum* in Experiment 1 was 0.17 ± 0.02 mg g^-1^, and showed limited variation among populations (Fig. 6). In response to the addition of 1.0 mM Al^3+^ to the nutrient solution mean foliar Al concentration increased to values in the range 2.8 ± 0.5 to 10.5 ± 2.8 mg g^−1^. A PCA based on population mean values of foliar concentrations of Al, P, K, Ca and Mg for seedlings grown with Al addition uncovered a first axis explaining 71.6 % of the variation in the data and a second axis explaining 20.0 % of variation (Fig. 7; **see ****Supporting Information—Table S10**). The first axis of this PCA was correlated positively with foliar concentrations of all five elements, and with mean RGR of *M. malabathricum* populations under Al addition (*r* = 0.52, *P* < 0.041).

## Discussion

### Growth responses to Al addition

This study confirms that growth of the Al accumulator *M. malabathricum* is stimulated by the presence of low concentrations of Al in the growth medium, but we extend previous research by showing that the magnitude of this response differs among populations of this Al accumulator. Faster growth rate among populations was associated with increased tolerance to higher external Al concentrations and to higher foliar concentrations of Al, P, K, Ca and Mg, which may have been supported by increased allocation to root and stem biomass in response to Al addition.

*M. malabathricum* seedlings grown in the presence of Al in the nutrient solution doubled in dry mass over 28 days relative to seedlings grown in the absence of Al and displayed 14 % greater relative growth rate over a similar interval. These findings are consistent with positive growth responses to Al addition in previous studies on *M. malabathricum* (Osaki *et al.* 1997; Watanabe *et al.* 2005; Metali 2010) and other Al accumulators such as tea (*Camelia sinensis*) (Konishi *et al.* 1985; Fung *et al.* 2008; Morita *et al.* 2008; Hajiboland *et al.* 2013; Tolrà *et al.* 2020), as well non Al accumulators such as *Miscanthus sinensis* (Yoshii 1937) and *Eucalyptus gummifera* (Mullette 1975). In our experiments with *M. malabathricum*, the beneficial effect of Al on growth occurred when Al was supplied at concentrations of 0.5 mM, 1.0 mM and 2.0 mM Al in the nutrient solutions, which is similar to the evidence showing growth stimulation of tea seedlings in nutrient solutions containing 0.25 mM and 0.5 mM Al (Konishi *et al.* 1985; Fung *et al.* 2008).

Several mechanisms have been proposed to explain this positive growth response to Al addition. Recent suggestions are that Al ameliorates H^+^ toxicity for plants growing in acid soils (Osaki *et al.* 1997; Kidd and Proctor 2001; Delhaize *et al.* 2012) or that Al alleviates Fe toxicity (Watanabe *et al.* 2005; Hajiboland *et al.* 2013; Poschenrieder *et al.* 2015). The general symptoms of Fe toxicity include inhibition of leaf function and a reduction in photosynthetic rate (Kampfenkel *et al.* 1995), arising from oxidative stress and disruption of membrane functions associated with lipid peroxidation and lignin deposition in roots (Watanabe *et al.* 2006). Evidence in favour of this hypothesis was provided by Hajiboland *et al.* (2013) who showed that Fe concentrations were reduced in the roots and leaves of tea plants grown hydroponically with the addition of 200 µM Al, which then led to the stimulation of tea growth. There is a suggestion that *M. malabathricum* expresses symptoms of Fe toxicity in response to low concentrations (i.e. 40 µm) of Fe in nutrient solutions, and that Al addition reduces tissue Fe concentrations and relieves these symptoms (Watanabe *et al.* 2006). The Fe concentration in the nutrient solution used in our experiments (20 µm) was closer to the Fe concentration treatment for which Al addition did not relieve symptoms of Fe toxicity in *M. malabathricum* (10 µm) than the treatment that did so (100 µm) in Watanabe *et al.* (2006), and we observed no symptoms of Fe toxicity in seedlings in the presence or absence of Al addition. However, it remains possible that interactions between Al and Fe contributed to the growth response to Al addition that we observed.

A second potential mechanism for growth stimulation by Al addition arises from the observation that Al addition in low concentrations increases photosynthetic rates as well as the length and surface area of roots in tea plants (Hajiboland *et al.* 2013). These changes may facilitate enhanced uptake of limiting soil nutrients and thus contribute to the growth response (Mukhopadyay *et al.* 2012; Hajiboland *et al.* 2013). This interpretation is consistent with our earlier finding that Al addition increased concentrations of P, K, Ca and Mg in *M. malabathricum* seedlings (Khairil and Burslem 2018) and the results reported in this paper showing that populations expressing a higher growth rate in response to Al addition had higher foliar concentrations of these nutrients.

The results of the first experiment suggested that growth rates varied among the 18 populations, but they had the same magnitude of response to Al addition. However, this absence of evidence for an interaction between population and Al treatment is likely to reflect the low statistical power of this experiment, which was based on only three replicates per population and only two Al treatments. This was examined in greater depth in the second experiment, in which replication was doubled and the number of Al treatments increased to five across a range of concentrations from 0 to 5 mM. The increased resolution of this experiment uncovered a significant interaction between population and Al treatment showing that the faster-growing population had a greater magnitude of response to Al addition and a requirement for higher Al concentrations in the growth medium to achieve maximum growth rates. These findings reinforce the conclusion that inherent differences in growth rate among populations are linked to responsiveness to Al supply. Population-level differentiation in seedling growth responses to Al addition has not been addressed previously, but is likely to arise from variation in the genetic factors underlying the expression of Al accumulation and phenotypic plasticity in the traits that determine growth rate (Pressoir and Berthaud 2004; Andrew *et al.* 2010). For *M. malabathricum*, our study shows that variation in growth rate among populations was positively correlated with increasing foliar Al and nutrient concentrations, while there was no evidence that differentiation in biomass allocation was directly responsible for differential growth rates. These results suggest that the physiological determinants of variation in growth rate and Al accumulation among populations are linked to enhanced nutrient capture, which supports similar evidence obtained using a single population of *M. malabathricum* (Watanabe *et al.* 1997, 2006; Watanabe and Osaki 2001) as well as other plant species (Ghanati *et al.* 2005; Fung *et al.* 2008; Hajiboland *et al.* 2013; Tolra *et al.* 2020). Although genetic differentiation among populations of Al accumulators has not been studied, evidence from studies of other metal hyper-accumulators is available (Assunção *et al.* 2003, 2008; Andrew *et al.* 2010; Halimaa *et al.* 2014). For example, genetic variation among populations of *Thlaspi caerulescens* contributes to their differential capacity to accumulate and tolerate soil Zn and Cd concentrations (Assunção *et al.* 2003, 2008; Halimaa *et al.* 2014). Further research is required to determine how genetic variation contributes to co-varying physiological differences in Al accumulation, growth rate and nutrient uptake among populations of *M. malabathricum* and other Al accumulators.

Responses to Al addition in terms of biomass allocation provide important insights into the physiological mechanisms regulating the stimulation of growth rate in *M. malabathricum*. Al addition resulted in increases in biomass allocation to roots (2.4 %) and stems (1.0 %) and a decrease in mass allocation to leaves (−3.4 %). Other studies on *M. malabathricum* have also demonstrated that Al addition results in diversion of dry mass to roots, as well as increases in root activity, root elongation, expansion of fine roots and activation of citrate synthase in roots (Watanabe *et al.* 2005, Metali 2010). This expansion of root mass and root surface area may help to explain the stimulation of nutrient uptake by Al addition in Al accumulators including both *M. malabathricum* and tea (Ghanati *et al.* 2005; Hajiboland *et al.* 2013; Khairil and Burslem 2018). Similarly, Al addition induces elongation of central cap cells and root elongation in tea plants (Konishi *et al.* 1985; Fung *et al.* 2008). These uniform patterns of growth stimulation and modification of biomass distribution in response to the addition of similar low concentrations of Al in two highly dissimilar species of Al accumulator suggests that there may be a common physiological mechanism underlying the response.

For the expansion of root systems to occur alongside a stimulation of growth in *M. malabathricum* there must be compensatory increases in carbon assimilation rate per unit of photosynthetic leaf mass. This was not examined in our study, but Metali (2010) reported that an increase in growth in response to Al addition was associated with substantial increases in net assimilation rate (NAR) and smaller increases in specific leaf area (SLA) in *M. malabathricum*, which supports this prediction. Research on tea has also revealed a positive effect of Al addition on rates of photosynthesis (Ghanati *et al.* 2005; Hajiboland *et al.* 2013), which would explain the increase in net assimilation rate per unit leaf area if a similar response occurred in *M. malabathricum*.

### Al toxicity at high concentrations

Seedlings of both populations of *M. malabathricum* displayed retarded growth at a concentration of 5.0 mM Al in the nutrient solution, which suggests that they were experiencing Al toxicity symptoms at this concentration, whereas growth was stimulated at a concentration of 2.0 mM Al in the nutrient solution. Therefore, *M. malabathricum* is more tolerant of high Al concentrations than tea, which displays reduction of growth at Al concentrations in the medium greater than 1.0 mM and defoliation at concentrations of 5.0 and 10 mM Al (Fung *et al.* 2008; Mukhopadyay *et al.* 2012). Growth reductions at high Al concentrations in tea may be caused by rhizotoxicity, where Al binds to plasma membranes in cell walls of the sensitive root apex zone (Kochian *et al.* 2004; Fung *et al.* 2008; Horst *et al.* 2010; Mukhopadyay *et al.* 2012). High Al concentrations in the rhizosphere may also cause a reduction in nutrient uptake through effects on the net extrusion of H^+^ by plasma membrane ATPase leading to decreases in the loading of polyvalent cations (Rengel 1996; Poschenrieder *et al.* 2008). These studies suggest that the limit to tolerance of high Al by Al accumulators is determined by effects on root metabolism and nutrient uptake (Konishi *et al.* 1985; Fung *et al.* 2008; Horst *et al.* 2010; Mukhopadyay *et al.* 2012).

### Al accumulation in *M. malabathricum*

The status of *M. malabathricum* as an Al accumulator plant was supported by this study (Chenery 1948; Jansen 2002; Watanabe *et al.* 2005, 2008) and we build on this finding by demonstrating significant inter-population differences in the magnitude of Al accumulation and the linkages among Al accumulation, growth rate and foliar nutrient concentrations. Our previous research has shown that for *M. malabathricum* seedlings grown in nutrient solutions with Al, foliar Al concentration was positively correlated with foliar Ca, K and Mg concentrations among the same 18 populations examined here (Khairil and Burslem 2018). This finding is consistent with studies showing increased uptake of P, Ca, K and Mg concentrations in response to Al addition in both *M. malabathricum* (Watanabe *et al*. 2005, Metali 2010, Khairil and Burslem 2018) and tea (Fung *et al.* 2008). The consistent pattern of association among these foliar elements for multiple populations and species of Al accumulators suggests that there may be a common underlying uptake mechanism for these elements (Masunaga *et al.* 1998; Metali *et al.* 2015). The relationship between the single strong axis of variation in foliar nutrient concentrations to seedling growth rate reported in this paper suggests that the Al-induced stimulation of both nutrient uptake and growth rate may be physiologically coupled in *M. malabathricum*.

## Conclusions

We conclude that populations of the Al accumulator *M. malabathricum* have adapted to express a physiological response to Al concentration in the growth medium, which leads to a stimulation of growth. The growth stimulation is associated with enhanced uptake of nutrients including P, Ca, K and Mg, which differed among populations of this species and may have a genetic basis. Al addition triggered an enhanced allocation of dry mass to roots at the expense of leaves, which must be coupled with faster rates of carbon assimilation per unit leaf mass in order to generate a growth response to Al addition.

## Supporting Information

The following additional information is available in the online version of this article —

**Table S1.** Mean (±SE) foliar Al concentration (mg g^−1^) in the Al+ treatment, relative growth rate (RGR, day^−1^) in Al+ and Al− treatments, difference in mean RGR between treatments (day^−1^) and growth stimulation in the Al+ treatment (as a % of the Al− treatment) for 18 *Melastoma malabathricum* populations with 1.0 mM AlCl_3_ (Al+ treatment) or without Al addition (Al−) in the nutrient solution. The populations are ranked based on foliar Al concentration in the Al+ treatment.

**Table S2.** Mean square values (MS), *F* statistics and *P* values following two-way analysis of variance (ANOVA) to determine the significance of differences among populations (Population), Al treatments (Treatment) and the interaction between population and Al treatment on the dry mass of roots, stems, leaves and whole plants for seedlings of 18 populations of *M. malabathricum* grown with and without Al application. The significance of these values is indicated as follows: *, *P* < 0.05; **, *P* < 0.01; ***, *P* < 0.001.

**Table S3.** Mean square values (MS), *F* statistics and *P* values following two-way analysis of variance (ANOVA) to determine the significance of differences among populations (Population), Al treatments (Treatment) and the interaction between population and Al treatment on relative growth rate (RGR) of roots, stems, leaves and whole plants for seedlings of 18 populations of *M. malabathricum* grown with and without Al application. The significance of these values is indicated as follows: *, *P* < 0.05; **, *P* < 0.01; ***, *P* < 0.001.

**Table S4.** Mean square values (MS), *F* statistics and *P* values following two-way analysis of variance (ANOVA) to determine the significance of differences among populations (Population), Al treatments (Treatment) and the interaction between population and Al treatment on the dry mass of roots, stems, leaves and whole plants for seedlings of slow-growing and fast-growing populations of *M. malabathricum* grown for 28 days in nutrient solutions containing 0 mM, 0.5 mM, 1.0 mM, 2.0 mM or 5.0 mM AlCl_3_. The significance of these values is indicated as follows: *, *P* < 0.05; **, *P* < 0.01; ***, *P* < 0.001.

**Table S5.** Mean square values (MS), *F* statistics and *P* values following two-way analysis of variance (ANOVA) to determine the significance of differences among populations (Population), Al treatments (Treatment) and the interaction between population and Al treatment on the relative growth rate (RGR) of roots, stems, leaves and whole plants for seedlings of slow-growing and fast-growing populations of *M. malabathricum* grown for 28 days in nutrient solutions containing 0 mM, 0.5 mM, 1.0 mM, 2.0 mM or 5.0 mM AlCl_3_. The significance of these values is indicated as follows: *, *P* < 0.05; **, *P* < 0.01; ***, *P* < 0.001.

**Table S6.** Mean square values (MS), *F* statistics and *P* values following two-way analysis of variance (ANOVA) to determine the significance of differences among populations (Population), Al treatments (Treatment) and the interaction between population and Al treatment on root mass ratio (RMR), stem mass ratio (SMR) and leaf mass ratio (LMR) for seedlings of 18 populations of *M. malabathricum* grown with and without Al application. The significance of these values is indicated as follows: *, *P* < 0.05; **, *P* < 0.01; ***, *P* <0.001.

**Table S7.** Mean square values (MS), *F* statistics and *P* values following two-way analysis of variance (ANOVA) to determine the significance of differences among populations (Population), Al treatments (Treatment) and the interaction between population and Al treatment on root mass ratio (RMR), stem mass ratio (SMR) and leaf mass ratio (LMR) for seedlings of slow-growing and fast-growing populations of *M. malabathricum* grown for 28 days in nutrient solutions containing 0 mM, 0.5 mM, 1.0 mM, 2.0 mM or 5.0 mM AlCl_3._ The significance of these values is indicated as follows: *, *P* < 0.05; **, *P* < 0.01; ***, *P* < 0.001.

**Table S8.** Results from a principal components analysis (PCA) summarizing variation in biomass allocation among seedlings derived from 18 populations of *M. malabathricum* grown for 28 days with Al addition.

**Table S9.** Results from a principal components analysis (PCA) summarizing variation in biomass allocation among seedlings derived from 18 populations of *M. malabathricum* grown without Al addition.

**Table S10.** Results from a principal components analysis (PCA) summarizing variation in foliar concentrations among seedlings derived from 18 populations of *M. malabathricum* grown for 28 days with Al addition.

**Figure S1.** Locations of the 18 *Melastoma malabathricum* populations sampled for this study.

**Figure S2.** Boxplots of leaf mass ratio (LMR) of *M. malabathricum* seedlings of the slow-growing population (white panel) and fast-growing population (dark panel) after growth for 28 days in nutrient solutions containing 0 mM, 0.5 mM, 1.0 mM, 2.0 mM and 5.0 mM AlCl_3_.

**Figure S3.** Boxplots of stem mass ratio (SMR) of *M. malabathricum* seedlings of the slow-growing population (white panel) and fast-growing population (dark panel) after growth for 28 days in nutrient solutions containing 0 mM, 0.5 mM, 1.0 mM, 2.0 mM and 5.0 mM AlCl_3_.

**Figure S4.** Boxplots of root mass ratio (RMR) of *M. malabathricum* seedlings of the slow-growing population (white panel) and fast-growing population (dark panel) after growth for 28 days in nutrient solutions containing 0 mM, 0.5 mM, 1.0 mM, 2.0 mM and 5.0 mM AlCl_3_.

plaa049_suppl_Supplementary_Material

## Data Availability

The data underlying this study are published as open access at the *Drayd*.org (https://doi.org/10.5061/dryad.gqnk98sk7)

## Notes

**Form & Function. Chief Editor:** Kate McCulloh

## References

[CIT0001] Andrew RL, Wallis IR, Harwood CE, Foley WJ 2010 Genetic and environmental contributions to variation and population divergence in a broad-spectrum foliar defence of *Eucalyptus tricarpa*. Annals of Botany 105:707–717. 20228089 10.1093/aob/mcq034 PMC2859910

[CIT0002] Assunção AGL, Bookum WM, Nelissen HJM, Vooijs R, Schat H, Ernst WHO 2003 Differential metal-specific tolerance and accumulation patterns among *Thlaspi caerulescens* populations originating from different soil types. New Phytologist 159:411–419. 10.1046/j.1469-8137.2003.00819.x 33873347

[CIT0054] Assunção aGL, Bleeker P, ten Bookum WM, Vooijs R, Schat H 2008 Intraspecific variation of metal preference patterns for hyperaccumulation in *Thlaspi caerulescens:* evidence from binary metal exposures. Plant and Soil 303:289–299.

[CIT0003] Barceló J, Poschenrieder C 2002 Fast root growth responses, root exudates, and internal detoxification as clues to the mechanisms of aluminium toxicity and resistance: a review. Environmental and Experimental Botany 48:75–92. doi:10.1016/S0098-8472(02)00013-8

[CIT0004] Chenery EM 1948 Aluminium in the plant world author. Royal Botanic Gardens 3:173–183.

[CIT0005] Clark RB 1977 Effect of aluminium on growth and mineral elements of Al-tolerant and Al-intolerant corn. Plant and Soil 2:653–662.

[CIT0006] Delhaize E, Ma JF, Ryan PR 2012 Transcriptional regulation of aluminium tolerance genes. Trends in Plant Science 17:341–348. 22459757 10.1016/j.tplants.2012.02.008

[CIT0007] Doncheva S 2005 Root cell patterning: a primary target for aluminium toxicity in maize. Journal of Experimental Botany 56:1213–1220. doi:10.1093/jxb/eri115 15737983

[CIT0008] Fung KF, Carr HP, Zhang J, Wong MH 2008 Growth and nutrient uptake of tea under different aluminium concentrations. Journal of Science of Food and Agriculture 1591:1582–1591. doi:10.1002/jsfa

[CIT0009] Ghanati F, Morita A, Yokota H 2005 Effects of aluminum on the growth of tea plant and activation of antioxidant system. Plant and Soil 276: 133–141. doi:10.1007/s11104-005-3697-y

[CIT0010] Godbold DL, Fritz E, Hüttermann A 1988 Aluminum toxicity and forest decline. Proceedings of the National Academy of Sciences of the United States of America 85:3888–3892. 16593936 10.1073/pnas.85.11.3888 PMC280325

[CIT0011] Hajiboland R, Bahrami Rad S, Barcelo J, Poschenrieder C 2013 Mechanisms of aluminum-induced growth stimulation in tea (*Camellia sinensis*). Journal of Plant Nutrition and Soil Science 176:616–625. doi:10.1002/jpln.201200311

[CIT0012] Halimaa P, Blande D, Aarts MG, Tuomainen M, Tervahauta A, Kärenlampi S 2014 Comparative transcriptome analysis of the metal hyperaccumulator *Noccaea caerulescens*. Frontiers in Plant Science 5:213. 24904610 10.3389/fpls.2014.00213 PMC4033236

[CIT0013] Horst WJ, Wang Y, Eticha D 2010 The role of the root apoplast in aluminium-induced inhibition of root elongation and in aluminium resistance of plants: a review. Annals of Botany 106:185–197. 20237112 10.1093/aob/mcq053 PMC2889789

[CIT0014] Hunt R 1982 Plant growth curves: the functional approach to plant growth analysis. London, UK: Edward Arnold. doi:10.1093/aob/mcf214

[CIT0015] Jansen S 2002 Aluminum hyperaccumulation in angiosperms : a review of its phylogenetic significance. The Botanical Review 68:235–269.

[CIT0016] Jansen S 2003 A comparative study of metal levels in leaves of some Al-accumulating Rubiaceae. Annals of Botany 91:657–663. doi:10.1093/aob/mcg071 12714364 PMC4242354

[CIT0017] Jansen S, Watanabe T, Smets E 2002 Aluminium accumulation in leaves of 127 species in Melastomataceae, with comments on the order Myrtales. Annals of Botany 90:53–64. 12125773 10.1093/aob/mcf142 PMC4233848

[CIT0018] Joffry SM, Yob NJ, Rofiee MS, Affandi MM, Suhaili Z, Othman F, Akim AM, Desa MN, Zakaria ZA 2012 *Melastoma malabathricum* (L.) Smith ethnomedicinal uses, chemical constituents, and pharmacological properties: a review. Evidence-Based Complementary and Alternative Medicine: Ecam 2012:258434. 22242040 10.1155/2012/258434 PMC3254175

[CIT0019] Kampfenkel K, Van Montagu M, Inze D 1995 Effects of iron excess on nicotiana plumbaginifolia plants (implications to oxidative stress). Plant Physiology 107:725–735. 12228397 10.1104/pp.107.3.725 PMC157188

[CIT0020] Khairil M, Burslem DFRP 2018 Controls on foliar aluminium accumulation among populations of the tropical shrub *Melastoma malabathricum* L. (Melastomataceae). Tree Physiology 38:1752–1760. 30137635 10.1093/treephys/tpy082

[CIT0021] Kidd PS, Proctor J 2001 Why plants grow poorly on very acid soils: are ecologists missing the obvious? Journal of Experimental Botany 52: 791–799. doi:10.1093/jexbot/52.357.791 11413215

[CIT0022] Kochian LV, Hoekenga OA, Pineros MA 2004 How do crop plants tolerate acid soils? Mechanisms of aluminum tolerance and phosphorous efficiency. Annual Review of Plant Biology 55:459–493. 10.1146/annurev.arplant.55.031903.141655 15377228

[CIT0023] Konishi S, Miyamoto S, Taki T 1985 Stimulatory effects of aluminum on tea plants grown under low and high phosphorus supply. Soil Science and Plant Nutrition 31:361–368. doi:10.1080/00380768.1985.10557443

[CIT0024] Masunaga T, Kubota D, Hotta M, Wakatsuki T 1998 Mineral composition of leaves and bark in aluminum accumulators in a tropical rain forest in Indonesia. Soil Science and Plant Nutrition 44:347–358. doi:10.1080/00380768.1998.10414456

[CIT0025] Metali FH 2010 Factors controlling Al accumulation in plants : effects of phylogeny, soil conditions and external nutrient supply. PhD Thesis, University of Aberdeen, UK.

[CIT0026] Metali F, Abu Salim K, Tennakoon K, Burslem DF 2015 Controls on foliar nutrient and aluminium concentrations in a tropical tree flora: phylogeny, soil chemistry and interactions among elements. The New Phytologist 205:280–292. 25138655 10.1111/nph.12987

[CIT0027] Metali F, Salim KA, Burslem DF 2012 Evidence of foliar aluminium accumulation in local, regional and global datasets of wild plants. The New Phytologist 193:637–649. 22111583 10.1111/j.1469-8137.2011.03965.x

[CIT0028] Morita A, Yanagisawa O, Takatsu S, Maeda S, Hiradate S 2008 Mechanism for the detoxification of aluminum in roots of tea plant (*Camellia sinensis* (L.) Kuntze). Phytochemistry 69:147–153. 17643454 10.1016/j.phytochem.2007.06.007

[CIT0029] Mukhopadyay M, Bantawa P, Das A, Sarkar B, Bera B, Ghosh P, Mondal TK 2012 Changes of growth, photosynthesis and alteration of leaf antioxidative defence system of tea [*Camellia sinensis* (L.) O. Kuntze] seedlings under aluminum stress. Biometals: An International Journal on the Role of Metal Ions in Biology, Biochemistry, and Medicine 25:1141–1154. 10.1007/s10534-012-9576-0 22850809

[CIT0030] Mullette KJ 1975 Stimulation of growth in *Eucalyptus* due to aluminium. Plant and Soil 42:495–499. doi:10.1007/BF00010026

[CIT0031] Osaki M, Watanabe T, Ishizawa T 2003 Nutritional characteristics of the leaves of native plants. Plant Foods for Human Nutrition 58:93–115. 12906350 10.1023/a:1024415203690

[CIT0032] Osaki M, Watanabe T, Tadano T 1997 Beneficial effect of aluminum on growth of plants adapted to low pH soils. Soil Science and Plant Nutrition 43:551–563.

[CIT0033] Poschenrieder C, Gunsé B, Corrales I, Barceló J 2008 A glance into aluminum toxicity and resistance in plants. The Science of the Total Environment 400:356–368. 18657304 10.1016/j.scitotenv.2008.06.003

[CIT0034] Poschenrieder C, TBarcelo J, Hajiboland R, Arroyave C 2015 *Mechanism of hyper-resistance and hyper-tolerance to aluminium in plants*, Vol. 24. Panda S and Baluska F, eds. Switzerland: Springer International Publishing. doi:10.1007/978-3-319-19968-9

[CIT0035] Pressoir G, Berthaud J 2004 Population structure and strong divergent selection shape phenotypic diversification in maize landraces. *Heredity* 92:95–101. 14666128 10.1038/sj.hdy.6800388

[CIT0053] R Development Core Team 2016 R: a language and environment for statistical computing. Vienna, Austria: R Foundation for Statistical Computing.

[CIT0036] R’bia O, Horchani F, Smida I, Mejri M, Aschi-Smiti S 2011 Aluminium phytotoxicity and plant acclimation to acidic soils. International Journal of Agricultural Research, 6:194–208. doi:10.3923/ijar.2011.194.208

[CIT0037] Rascio N, Navari-Izzo F 2011 Heavy metal hyperaccumulating plants: how and why do they do it? And what makes them so interesting? Plant Science: An International Journal of Experimental Plant Biology 180:169–181. 21421358 10.1016/j.plantsci.2010.08.016

[CIT0038] Rengel Z 1996 Tansley review no 89—Uptake of aluminium by plant cells. New Phytologist 134:389–406.

[CIT0039] Ryan PR, Ditomaso JM, Kochian LV 1993 Aluminium toxicity in roots: an investigation of spatial sensitivity and the role of the root cap. Journal of Experimental Botany 44:437–446. doi:10.1093/jxb/44.2.437

[CIT0040] Schmitt M, Boras S, Tjoa A, Watanabe T, Jansen S 2016 Aluminium accumulation and intra-tree distribution patterns in three *Arbor aluminosa* (Symplocos) species from central Sulawesi. Plos ONE 11:e0149078. 26871698 10.1371/journal.pone.0149078 PMC4752314

[CIT0041] Sharma HK, Chhangte L, Dolui AK 2001 Traditional medicinal plants in Mizoram, India. Fitoterapia 72:146–161. 11223224 10.1016/s0367-326x(00)00278-1

[CIT0042] de Souza MC, Habermann G, Amaral CL, Rosa AL, Pinheiro MHO, Da Costa FB 2017 *Vochysia tucanorum* Mart.: an aluminum-accumulating species evidencing calcifuge behavior. Plant Soil 419:377–389.

[CIT0043] de Souza MC, Williams TCR, Poschenrieder C, Jansen S, Pinheiro MHO, Soares IP, Franco AC 2020 Calcicole behaviour of *Callisthene fasciculata* Mart., an Al-accumulating species from the Brazilian Cerrado. Plant Biology (Stuttgart, Germany) 22:30–37. 10.1111/plb.13036 31368234

[CIT0044] Tolrà R, Martos S, Hajiboland R, Poschenrieder C 2020 Aluminium alters mineral composition and polyphenol metabolism in leaves of tea plants (*Camellia sinensis*). Journal of Inorganic Biochemistry 204:110956. 31862583 10.1016/j.jinorgbio.2019.110956

[CIT0045] Watanabe T, Jansen S, Osaki M 2005 The beneficial effect of aluminium and the role of citrate in Al accumulation in *Melastoma malabathricum*. The New Phytologist 165:773–780. 15720688 10.1111/j.1469-8137.2004.01261.x

[CIT0046] Watanabe T, Jansen S, Osaki M 2006 Al–Fe interactions and growth enhancement in *Melastoma malabathricum* and *Miscanthus sinensis* dominating acid sulphate soils. Plant, Cell and Environment 29: 2124–2132. doi:10.1111/j.1365-3040.2006.01586.x 17081246

[CIT0047] Watanabe T, Osaki M 2001 Influence of aluminum and phosphorus on growth and xylem sap composition in *Melastoma malabathricum* L. Plant and Soil 237:63–70. doi:10.1023/A:1013395814958

[CIT0048] Watanabe T, Osaki M 2002 Mechanisms of adaptation to high aluminum condition in native plant species growing in acid soils: a review. Communications in Soil Science and Plant Analysis 33:1247–1260. doi:10.1081/CSS-120003885

[CIT0049] Watanabe T, Osaki M, Tadano T 1997 Aluminum-induced growth stimulation in relation to calcium, magnesium, and silicate nutrition in *Melastoma malabathricum* L. Soil Science and Plant Nutrition 43:827–837. doi:10.1080/00380768.1997.10414649

[CIT0050] Watanabe T, Osaki M, Yoshihara T, Tadano T 1998 Distribution and chemical speciation of aluminum in the Al accumulator plant, *Melastoma malabathricum* L. Plant and Soil 201:165–173.

[CIT0052] Watanabe T, Misawa S, Hiradate S, Osaki M 2008 Root mucilage enhances aluminum accumulation in *Melastoma malabathricum*, an aluminum accumulator. Plant Signaling & Behavior 3:603–605. 19704812 10.4161/psb.3.8.6356 PMC2634511

[CIT0051] Yoshii Y 1937 Aluminium requirements of solfatara-plants. The Botanical Magazine 1:262–270. doi:10.1007/s13398-014-0173-7.2

