# Supplementary material for: Contrasting growth responses to aluminium addition among populations of the aluminium accumulator Melastoma malabathricum
Source: AoB Plants. 2020 Sep 11;12(5):plaa049. doi: 10.1093/aobpla/plaa049 (PMC7750992; doi:10.1093/aobpla/plaa049)
Supplement: plaa049_suppl_Supplementary_Material [file plaa049_suppl_supplementary_material.pdf]

SUPPLEMENTARY INFORMATION

List of Figures and Tables

Figure S1. Locations of the 18 *Melastoma malabathricum* populations sampled for this study.

Figure S2. Boxplots of leaf mass ratio (LMR) of *M. malabathricum* seedlings of the slow-growing population (white panel) and fast-growing population (dark panel) after growth for 28 days in nutrient solutions containing 0 mM, 0.5 mM, 1.0 mM, 2.0 mM and 5.0 mM $AlCl_3$.

Figure S3. Boxplots of stem mass ratio (SMR) of *M. malabathricum* seedlings of the slow-growing population (white panel) and fast-growing population (dark panel) after growth for 28 days in nutrient solutions containing 0 mM, 0.5 mM, 1.0 mM, 2.0 mM and 5.0 mM $AlCl_3$.

Figure S4. Boxplots of root mass ratio (RMR) of *M. malabathricum* seedlings of the slow-growing population (white panel) and fast-growing population (dark panel) after growth for 28 days in nutrient solutions containing 0 mM, 0.5 mM, 1.0 mM, 2.0 mM and 5.0 mM $AlCl_3$.

TABLE S1. Mean (±SE) foliar Al concentration (mg g^{-1}) in the Al+ treatment, relative growth rate (RGR, d^{-1}) in Al+ and Al- treatments, difference in mean RGR between treatments (d^{-1}) and growth stimulation in the Al+ treatment (as a % of the Al- treatment) for 18 *Melastoma malabathricum* populations with 1.0 mM $AlCl_3$ (Al+ treatment) or without Al addition (Al-) in the nutrient solution. The populations are ranked based on foliar Al concentration in the Al+ treatment.

TABLE S2. Mean square values (MS), F Statistics and P values following two way analysis of variance (ANOVA) to determine the significance of differences among populations (Population), Al treatments (Treatment) and the interaction between population and Al treatment on the dry mass of roots, stems, leaves and whole plants for seedlings of 18 populations of *M. malabathricum* grown with and without Al application. The significance of these values is indicated as follow: *, $P < 0.05$; **, $P < 0.01$; ***, $P < 0.001$.

TABLE S3. Mean square values (MS), F Statistics and P values following two way analysis of variance (ANOVA) to determine the significance of differences among populations (Population), Al treatments (Treatment) and the interaction between population and Al treatment on relative growth rate (RGR) of roots, stems, leaves and whole plants for seedlings of 18 populations of *M. malabathricum* grown with and without Al application. The significance of these values is indicated as follow: *, $P < 0.05$; **, $P < 0.01$; ***, $P < 0.001$.

TABLE S4. Mean square values (MS), F Statistics and P values following two way analysis of variance (ANOVA) to determine the significance of differences among populations (Population), Al treatments (Treatment) and the interaction between population and Al treatment on the dry mass of roots, stems, leaves and whole plants for seedlings of slow-growing and fast-growing populations of *M. malabathricum* grown for 28 days in nutrient solutions containing 0 mM, 0.5 mM, 1.0 mM, 2.0mM or 5.0 mM $AlCl_3$. The significance of these values is indicated as follow: *, $P < 0.05$; **, $P < 0.01$; ***, $P < 0.001$.

TABLE S5. Mean square values (MS), F Statistics and P values following two way analysis of variance (ANOVA) to determine the significance of differences among populations (Population), Al treatments (Treatment) and the interaction between population and Al treatment on the relative growth rate (RGR) of roots, stems, leaves and whole plants for seedlings of slow-growing and fast-growing populations of *M. malabathricum* grown for 28 days in nutrient solutions containing 0 mM, 0.5 mM, 1.0 mM, 2.0mM or 5.0 mM $AlCl_3$. The significance of these values is indicated as follow: *, $P < 0.05$; **, $P < 0.01$; ***, $P < 0.001$.

TABLE S6. Mean square values (MS), F Statistics and P values following two way analysis of variance (ANOVA) to determine the significance of differences among populations (Population), Al treatments (Treatment) and the interaction between population and Al treatment on root mass ratio (RMR), stem mass ratio (SMR) and leaf mass ratio (LMR) for seedlings of 18 populations of *M. malabathricum* grown with and without Al application..

The significance of these values is indicated as follow: *, $P < 0.05$; **, $P < 0.01$; ***, $P < 0.001$.

TABLE S7. Mean square values (MS), F Statistics and P values following two way analysis of variance (ANOVA) to determine the significance of differences among populations (Population), Al treatments (Treatment) and the interaction between population and Al treatment on root mass ratio (RMR), stem mass ratio (SMR) and leaf mass ratio (LMR) for seedlings of slow-growing and fast-growing populations of *M. malabathricum* grown for 28 days in nutrient solutions containing 0 mM, 0.5 mM, 1.0 mM, 2.0mM or 5.0 mM $AlCl_3$. The significance of these values is indicated as follow: *, $P < 0.05$; **, $P < 0.01$; ***, $P < 0.001$.

TABLE S8. Results from a principal components analysis (PCA) summarising variation in biomass allocation among seedlings derived from 18 populations of *M. malabathricum* grown for 28 days with Al addition.

TABLE S9. Results from a principal components analysis (PCA) summarising variation in biomass allocation among seedlings derived from 18 populations of *M. malabathricum* grown without Al addition.

TABLE S10. Results from a principal components analysis (PCA) summarising variation in foliar concentrations among seedlings derived from 18 populations of *M. malabathricum* grown for 28 days with Al addition.

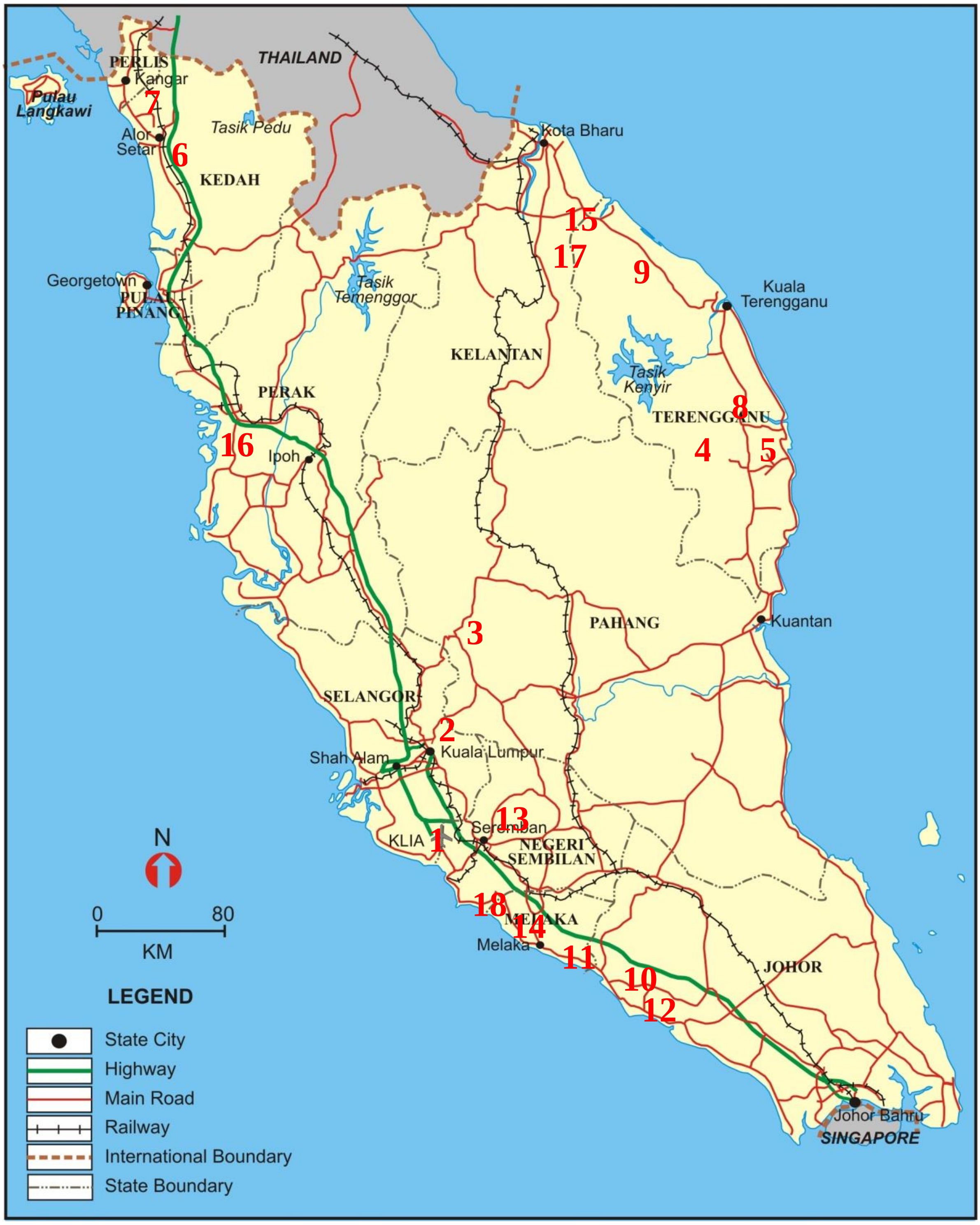

Fig. S1. Locations of the 18 *Melastoma malabathricum* populations sampled for this study.

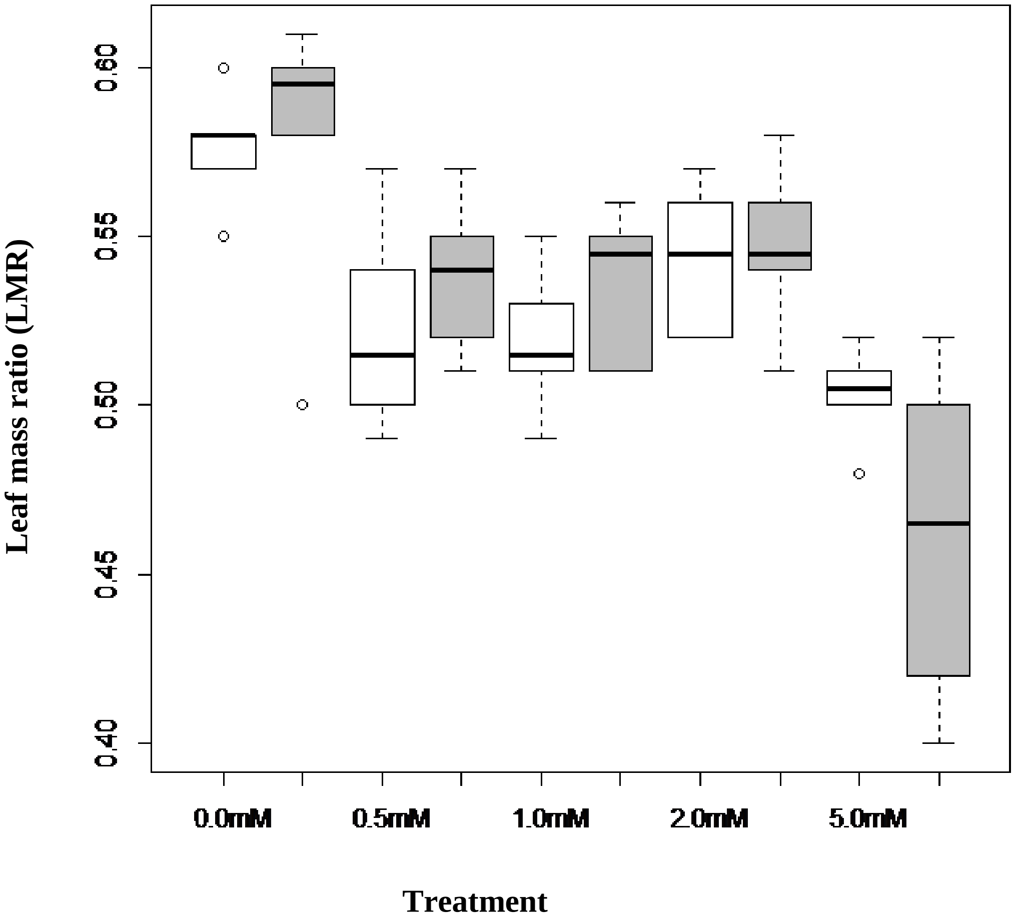

Fig. S2. Boxplots of leaf mass ratio (LMR) of *M. malabathricum* seedlings of the slow-growing population (white panel) and fast-growing population (dark panel) after growth for 28 days in nutrient solutions containing 0 mM, 0.5 mM, 1.0 mM, 2.0 mM and 5.0 mM $AlCl_3$.

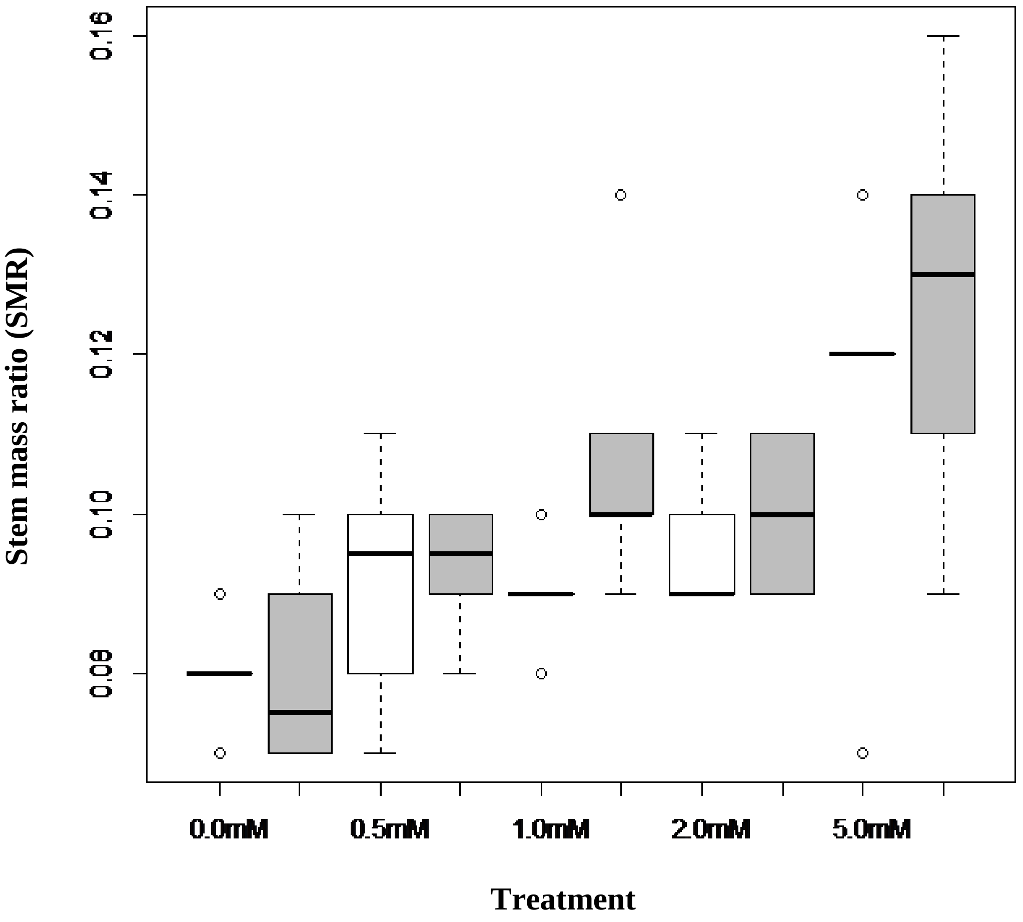

Fig. S3. Boxplots of stem mass ratio (SMR) of *M. malabathricum* seedlings of the slow-growing population (white panel) and fast-growing population (dark panel) after growth for 28 days in nutrient solutions containing 0 mM, 0.5 mM, 1.0 mM, 2.0 mM and 5.0 mM $AlCl_3$.

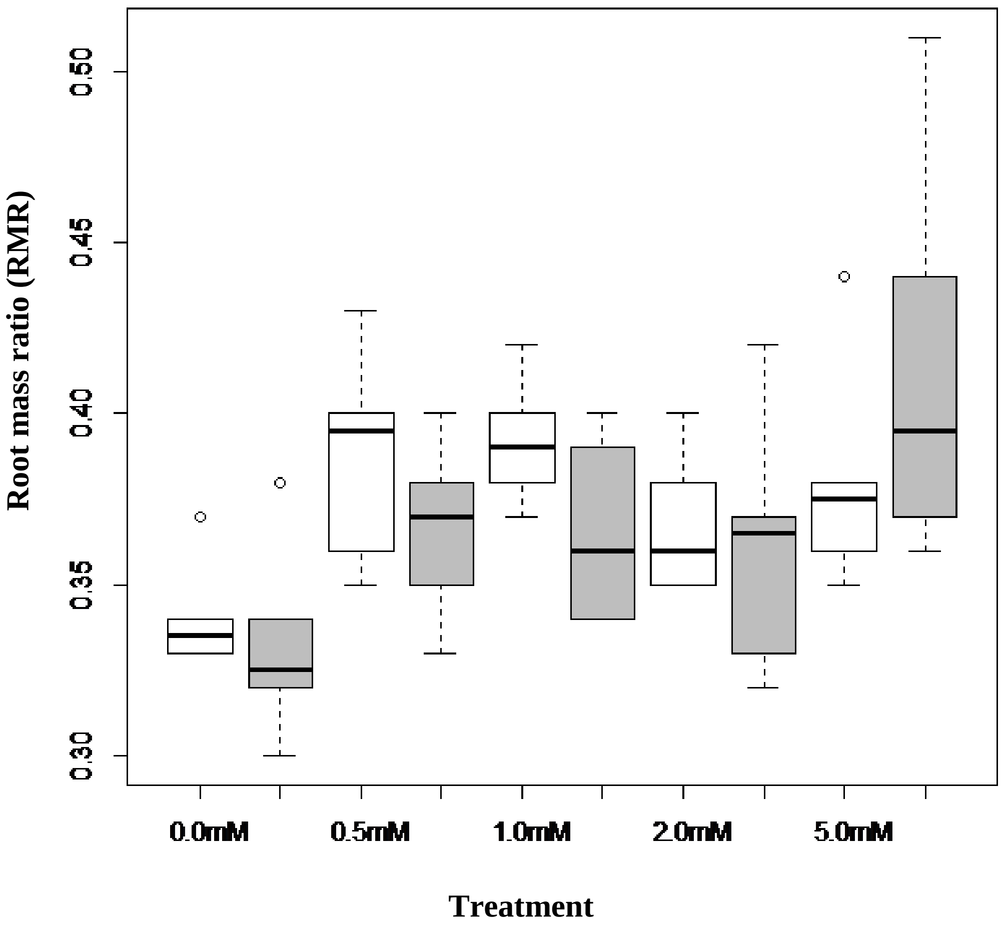

Fig. S4. Boxplots of root mass ratio (RMR) of *M. malabathricum* seedlings of the slow-growing population (white panel) and fast-growing population (dark panel) after growth for 28 days in nutrient solutions containing 0 mM, 0.5 mM, 1.0 mM, 2.0 mM and 5.0 mM $AlCl_3$.

Table S1. Mean (±SE) foliar Al concentration (mg g^{-1}) in the Al+ treatment, relative growth rate (RGR, d^{-1}) in Al+ and Al- treatments, difference in mean RGR between treatments (d^{-1}) and growth stimulation in the Al+ treatment (as a % of the Al- treatment) for 18 *Melastoma malabathricum* populations with 1.0 mM $AlCl_3$ (Al+ treatment) or without Al addition (Al-) in the nutrient solution. The populations are ranked based on foliar Al concentration in the Al+ treatment.

Pop	Rank	Mean foliar Al concentration (mg g^{-1})	Mean RGR Al+ treatment (d^{-1})	Mean RGR Al- treatment (d^{-1})	Difference in mean RGR (d^{-1})	Growth stimulation (%)
7	1	10.5±2.8	6.73±0.05	5.92±0.12	0.81	13.68
3	2	10.1±2.4	5.85±0.12	5.22±0.29	0.63	12.06
18	3	7.8±0.5	6.45±0.21	6.13±0.27	0.32	5.76
12	4	7.7±0.4	6.87±0.10	5.55±0.25	1.32	23.70
17	5	6.5±0.9	6.51±0.60	5.50±0.14	1.01	18.31
15	6	5.8±0.7	5.68±0.21	4.90±0.68	0.78	15.9
16	7	5.1±0.5	6.50±0.08	6.19±0.28	0.31	5.00
2	8	4.5±0.2	6.86±0.45	6.10±0.15	0.76	12.51
11	9	4.2±0.5	6.02±0.28	5.30±0.50	0.72	13.58
1	10	4.1±0.7	5.82±0.12	5.39±0.57	0.43	7.97
8	11	4.1±0.4	6.71±0.12	6.27±0.01	0.44	13.45
13	12	3.8±0.4	7.01±0.16	6.09±0.41	0.93	15.10
4	13	3.7±0.8	6.71±0.28	6.11±0.11	0.60	9.88
5	14	3.4±0.6	5.33±0.38	4.69±0.55	0.64	13.65
14	15	3.3±0.3	5.67±0.21	4.55±0.64	1.12	24.61
6	16	3.2±0.2	5.80±0.21	5.69±0.21	0.11	1.93
10	17	3.1±0.7	4.60±0.55	3.95±0.46	0.65	16.45
9	18	2.8±0.5	6.44±0.01	5.84±0.31	0.60	10.27

TABLE S2. Mean square values (MS), F Statistics and P values following two way analysis of variance (ANOVA) to determine the significance of differences among populations (Population), Al treatments (Treatment) and the interaction between population and Al treatment on the dry mass of roots, stems, leaves and whole plants for seedlings of 18 populations of *M. malabathricum* grown with and without Al application. The significance of these values is indicated as follow: *, P < 0.05; **, P < 0.01; ***, P < 0.001.

	Root Dry Mass				Stem Dry Mass			
Factors	df	MS	F Value	P Value	df	MS	F Value	P Value
Population	17	39563	6.461	2.85e-11 ***	17	3999	10.951	8.73e-16 ***
Treatment	1	620686	101.359	< 2e-16 ***	1	50628	113.917	< 2e-16 ***
Population: Treatment	17	18289	1.308	0.204474	17	1221	2.747	0.000544 ***
Residuals	150	6124			150	445		

	Leaf Dry Mass				Total Dry Mass			
Factors	df	MS	F Value	P Value	df	MS	F Value	P Value
Population	17	122852	7.441	4.79e-13 ***	17	363021	7.509	3.63e-13 ***
Treatment	1	962484	58.294	2.45e-12 ***	1	3975786	82.240	6.24e-16 ***
Population: Treatment	17	19390	1.174	0.292	17	1150906	1.400	0.144
Residuals	150	16511			150	48344		

TABLE S3. Mean square values (MS), F Statistics and P values following two way analysis of variance (ANOVA) to determine the significance of differences among populations (Population), Al treatments (Treatment) and the interaction between population and Al treatment on relative growth rate (RGR) of roots, stems, leaves and whole plants for seedlings of 18 populations of *M. malabathricum* grown with and without Al application. The significance of these values is indicated as follow: *, $P < 0.05$; **, $P < 0.01$; ***, $P < 0.001$.

	RGR Root (mg mg^{-1} d^{-1})				RGR Stem (mg mg^{-1} d^{-1})			
Factors	df	MS	F Value	P Value	df	MS	F Value	P Value
Population	17	3.090	6.123	1.21e-10 ***	17	3.64	6.519	2.23e-11 ***
Treatment	1	30.285	60.296	1.18e-12 ***	1	36.26	64.853	2.31e-13 ***
Population: Treatment	17	0.304	0.679	0.81624	17	0.391	0.797	0.69282

	RGR Leaf (mg mg^{-1} d^{-1})				Total RGR (mg mg^{-1} d^{-1})			
Factors	df	MS	F Value	P Value	df	MS	F Value	P Value
Population	17	4.148	6.861	5.26e-12 ***	17	3.605	6.103	5.11e-12 ***
Treatment	1	21.265	35.178	2.00e-08 ***	1	25.04	47.2	1.31e-10 ***
Population: Treatment	17	0.431	0.645	0.84717	17	0.255	0.285	0.977

TABLE S4. Mean square values (MS), F Statistics and P values following two way analysis of variance (ANOVA) to determine the significance of differences among populations (Population), Al treatments (Treatment) and the interaction between population and Al treatment on the dry mass of roots, stems, leaves and whole plants for seedlings of slow-growing and fast-growing populations of *M. malabathricum* grown for 28 days in nutrient solutions containing 0 mM, 0.5 mM, 1.0 mM, 2.0mM or 5.0 mM $AlCl_3$. The significance of these values is indicated as follow: *, P < 0.05; **, P < 0.01; ***, P < 0.001.

	Root Dry Mass				Stem Dry Mass			
Factors	df	MS	F Value	P Value	df	MS	F Value	P Value
Population	1	123365	24.806	<0.001 ***	1	15091	33.56	<0.001 ***
Treatment	4	139151	27.981	<0.001 ***	4	9808	21.81	<0.001 ***
Population: Treatment	4	17827	3.585	0.012 *	4	3085	6.86	<0.001 ***

	Leaf Dry Mass				Total Dry Mass			
Factors	df	MS	F Value	P Value	df	MS	F Value	P Value
Population	1	382319	31.578	<0.001 ***	1	1205289	31.104	<0.001 ***
Treatment	4	324997	26.843	<0.001 ***	4	1086850	28.047	<0.001 ***
Population: Treatment	4	54615	4.511	0.0034 **	4	175314	4.524	0.003 **

TABLE S5. Mean square values (MS), F Statistics and P values following two way analysis of variance (ANOVA) to determine the significance of differences among populations (Population), Al treatments (Treatment) and the interaction between population and Al treatment on the relative growth rate (RGR) of roots, stems, leaves and whole plants for seedlings of slow-growing and fast-growing populations of *M. malabathricum* grown for 28 days in nutrient solutions containing 0 mM, 0.5 mM, 1.0 mM, 2.0mM or 5.0 mM $AlCl_3$. The significance of these values is indicated as follow: *, P < 0.05; **, P < 0.01; ***, P < 0.001.

	RGR Root				RGR Stem			
Factors	df	MS	F Value	P Value	df	MS	F Value	P Value
Population	1	3.351	34.637	<0.001 ***	1	3.795	37.00	<0.001 ***
Treatment	4	6.877	71.075	<0.001 ***	4	6.534	63.701	<0.001 ***
Population: Treatment	4	0.763	7.891	<0.001 ***	4	0.391	3.809	0.008 **

	RGR Leaves				Total RGR			
Factors	df	MS	F Value	P Value	df	MS	F Value	P Value
Population	1	3.188	32.57	<0.001 ***	1	375055	10.845	<0.001 ***
Treatment	4	7.611	77.77	<0.001 ***	4	546549	15.804	<0.001 ***
Population: Treatment	4	0.241	2.46	0.0501*	4	60791	1.758	0.0063 **

TABLE S6. Mean square values (MS), F Statistics and P values following two way analysis of variance (ANOVA) to determine the significance of differences among populations (Population), Al treatments (Treatment) and the interaction between population and Al treatment on root mass ratio (RMR), stem mass ratio (SMR) and leaf mass ratio (LMR) for seedlings of 18 populations of *M. malabathricum* grown with and without Al application.. The significance of these values is indicated as follow: *, $P < 0.05$; **, $P < 0.01$; ***, $P < 0.001$.

	Root Mass Ratio (RMR)				Stem Mass Ratio (SMR)			
Factors	df	MS	F Value	P Value	df	MS	F Value	P Value
Population	17	0.010659	3.036	0.000145 ***	17	0.002402	9.820	1.58e-07 ***
Treatment	1	0.024549	6.992	0.009058 **	1	0.005226	4.513	0.00208 **
Population: Treatment	17	0.003077	0.876	0.602668	17	0.000644	1.210	0.26333

	Leaf Mass Ratio (LMR)			
Factors	df	MS	F Value	P Value
Population	17	0.01325	3.268	4.98e-05 ***
Treatment	1	0.05763	14.212	0.000234 ***
Population: Treatment	17	0.00366	0.902	0.572528

TABLE S7**.** Mean square values (MS), F Statistics and P values following two way analysis of variance (ANOVA) to determine the significance of differences among populations (Population), Al treatments (Treatment) and the interaction between population and Al treatment on root mass ratio (RMR), stem mass ratio (SMR) and leaf mass ratio (LMR) for seedlings of slow-growing and fast-growing populations of *M. malabathricum* grown for 28 days in nutrient solutions containing 0 mM, 0.5 mM, 1.0 mM, 2.0mM or 5.0 mM $AlCl_3$. The significance of these values is indicated as follow: *, P < 0.05; **, P < 0.01; ***, P < 0.001.

	Root mass ratio (RMR)				Stem mass ratio (SMR)			
Factors	df	MS	F Value	P Value	df	MS	F Value	P Value
Population	1	0.00028	0.019	0.891	1	0.0007350	3.472	0.0683
Treatment	4	0.014364	2.086	1.72e-0.5***	4	0.0026275	12.413	4.37e-07 ***
Population: Treatment	4	0.001779	8.867	0.063	4	0.0316	0.697	0.5977

	Leaf mass ratio (LMR)			
Factors	df	MS	F Value	P Value
Population	1	0.0021	3.432	0.0653
Treatment	4	0.0066	12.130	4.27e-07 ***
Population: Treatment	4	0.0831	0.667	0.5777

TABLE S8**.** Results from a principal components analysis (PCA) summarising variation in biomass allocation among seedlings derived from 18 populations of *M. malabathricum* grown for 28 days with Al addition. The table shows the standard deviation of each component, and the variation and cumulative variation explained by the three axes as well as the loadings of the three biomass allocation variables along the three axes.

Importance of components:	PC1	PC2	PC3
Standard deviation	1.414	0.991	0.132
Proportion of Variance	0.668	0.327	0.005
Cumulative Proportion	0.668	0.994	1.000
Loadings of biomass allocation			
RMR	0.704	0.014	-0.710
SMR	-0.322	0.897	-0.302
LMR	-0.633	-0.441	-0.636

TABLE S9**.** Results from a principal components analysis (PCA) summarising variation in biomass allocation among seedlings derived from 18 populations of *M. malabathricum* grown without Al addition. The table shows the standard deviation of each component, and the variation and cumulative variation explained by the three axes as well as the loadings of the three biomass allocation variables along the three axes.

Importance of components:	PC1	PC2	PC3
Standard deviation	1.533	0.802	0.082
Proportion of Variance	0.783	0.215	0.002
Cumulative Proportion	0.783	0.997	1.000
Loadings of biomass allocation			
RMR	0.574	-0.587	-0.570
SMR	0.498	0.804	-0.325
LMR	-0.649	0.097	-0.754

TABLE S10. Results from a principal components analysis (PCA) summarising variation in foliar concentrations among seedlings derived from 18 populations of *M. malabathricum* grown for 28 days with Al addition. The table shows the standard deviation of each component, and the variation and cumulative variation explained by the first three axes as well as the loadings of the five elements along the first three axes.

Importance of components:	PC1	PC2	PC3
Standard deviation	1.892	1.000	0.552
Proportion of Variance	0.716	0.200	0.061
Cumulative Proportion	0.716	0.916	0.977
Loadings of foliar concentrations along axes			
Al	0.455	-0.357	-0.605
P	0.287	0.499	0.498
K	0.311	-0.746	0.555
Ca	0.501	0.212	-0.274
Mg	0.512	0.148	0.054
